# Perceived Cost and Intrinsic Motor Variability Modulate the Speed-Accuracy Trade-Off

**DOI:** 10.1371/journal.pone.0139988

**Published:** 2015-10-08

**Authors:** Matteo Bertucco, Nasir H. Bhanpuri, Terence D. Sanger

**Affiliations:** 1 Department of Biomedical Engineering, University of Southern California, Los Angeles, California, United States of America; 2 Departments of Biomedical Engineering, Child Neurology, and Biokinesiology, University of Southern California and Children’s Hospital of Los Angeles, Los Angeles, California, United States of America; VU University Amsterdam, NETHERLANDS

## Abstract

Fitts’ Law describes the speed-accuracy trade-off of human movements, and it is an elegant strategy that compensates for random and uncontrollable noise in the motor system. The control strategy during targeted movements may also take into account the rewards or costs of any outcomes that may occur. The aim of this study was to test the hypothesis that movement time in Fitts’ Law emerges not only from the accuracy constraints of the task, but also depends on the perceived cost of error for missing the targets. Subjects were asked to touch targets on an iPad^®^ screen with different costs for missed targets. We manipulated the probability of error by comparing children with dystonia (who are characterized by increased intrinsic motor variability) to typically developing children. The results show a strong effect of the cost of error on the Fitts’ Law relationship characterized by an increase in movement time as cost increased. In addition, we observed a greater sensitivity to increased cost for children with dystonia, and this behavior appears to minimize the average cost. The findings support a proposed mathematical model that explains how movement time in a Fitts-like task is related to perceived risk.

## Introduction

Human movements are constantly performed in risky environments. Avoiding risk is so fundamental to natural movements that it is not surprising that behaviors change in different settings. When, for instance, we walk near the edge of a cliff or we move near delicate glass, there is a tendency to make smaller, slower, more cautious movements. We define the term “risk” to be the expected cost of behavior. Risk is thus the product of the probability of error and the cost of error [1–2]. A low cost but high probability of error is not risky, likewise, a high cost but low probability of error is not risky. For instance, walking on a very narrow wooden board suspended just a few centimeters above the floor, or walking across a safely enclosed bridge hundreds of meters above a canyon are both low-risk. It is only when high likelihood of error is combined with high cost of error that we face high-risk actions.

An important source of error is noise or unpredicted variability in movement. Experimental and computational studies have shown that multiple noise sources in the nervous system, from cellular to behavioral levels, contribute to trial-to-trial motor variability [3]. Moreover, the noise in motor commands tends to increase with the level of the motor command, known as signal-dependent noise [4–6]. Therefore, as the magnitude of motor commands increases, the variability of the movement increases, which consequently affects the probability of error of a particular action. The speed-accuracy trade-off of voluntary movements is a typical example of how the sensorimotor system may compensate for signal-dependent noise. Fitts’ Law is a widely known description of the mathematical relationship between movement speed and accuracy [7]. Traditionally, Fitts’ Law states that the movement time (MT) required to hit a target increases with movement distance (D) and decreases as target size increases (W), such that MT = a + b log_2_ (2D/W), wherein a and b are empirical constants, and log_2_(2D/W) represents the Index of Difficulty (ID). The inverse of slope 1/b is considered the Index of Performance, since the higher its value, the less MT is affected by increases in task difficulty (i.e. ID). If random and uncontrollable noise in the motor system increases as the movement speed increases, then increasing accuracy can only be achieved by decreasing the speed of movement [4]. In other words an increment of ID would decrease the movement speed in order to minimize the probability of missing a target. Thus, in the Fitts’ Law model, movement time is determined by the probability of error.

In recent years, planning and control of goal-oriented aiming movements have been described within the framework of decision-making under risk. In typical decision-making, the subject has to take into account not only the uncertainty of motor outcome after selecting the plan, but also the rewards or costs of any outcomes that may occur [8], [9–11]. For example, when different rewards are given related to movement endpoint variance, subjects tend to change their movement speed [12]. Some other elegant studies have shown that when participants perform pointing movements to a target with a penalty region around it, they shift their mean endpoints in response to changes in penalties and location of the penalty region relative to the target region [13–15]. Thus, these studies support the hypothesis that subjects can incorporate estimates of both the inherent motor variability and the cost of failure when planning and controlling fast goal-oriented aiming movements.

We thus hypothesized that movement time is determined not only by the accuracy required for the task, but also by the cost of errors. This requires a modification of Fitts’ Law to incorporate an additional term for cost, so that movement time depends on risk rather than just accuracy. The usual form of Fitts’ Law would arise in the situation where cost of error is constant, so that movement time would then vary only as a function of probability of error (determined by the relationship between speed and target size).

To test our hypothesis, subjects were asked to point at targets in a Fitts-like paradigm in which different costs were assigned for missing the target. We examined differences in intrinsic motor variability by comparing children with normal development and children affected by childhood dystonia. Childhood dystonia is a movement disorder in which involuntary sustained or intermittent muscle contractions cause twisting and repetitive movements, abnormal postures, or both [16]. In childhood dystonia there is augmented intrinsic variability, signal-dependent noise in motor execution, and abnormal tone [17, 18]. Therefore, we expected that increased motor variability would lead to higher sensitivity to the cost of error, slower movements, and decreased Index of Performance.

We propose a model that explains how movement time in a Fitts-like task is related to risk. We also compare our results to alternative models that incorporate cost of error in other plausible ways.

### A model of cost and probability of error in the speed-accuracy trade-off

**Fig 1:**
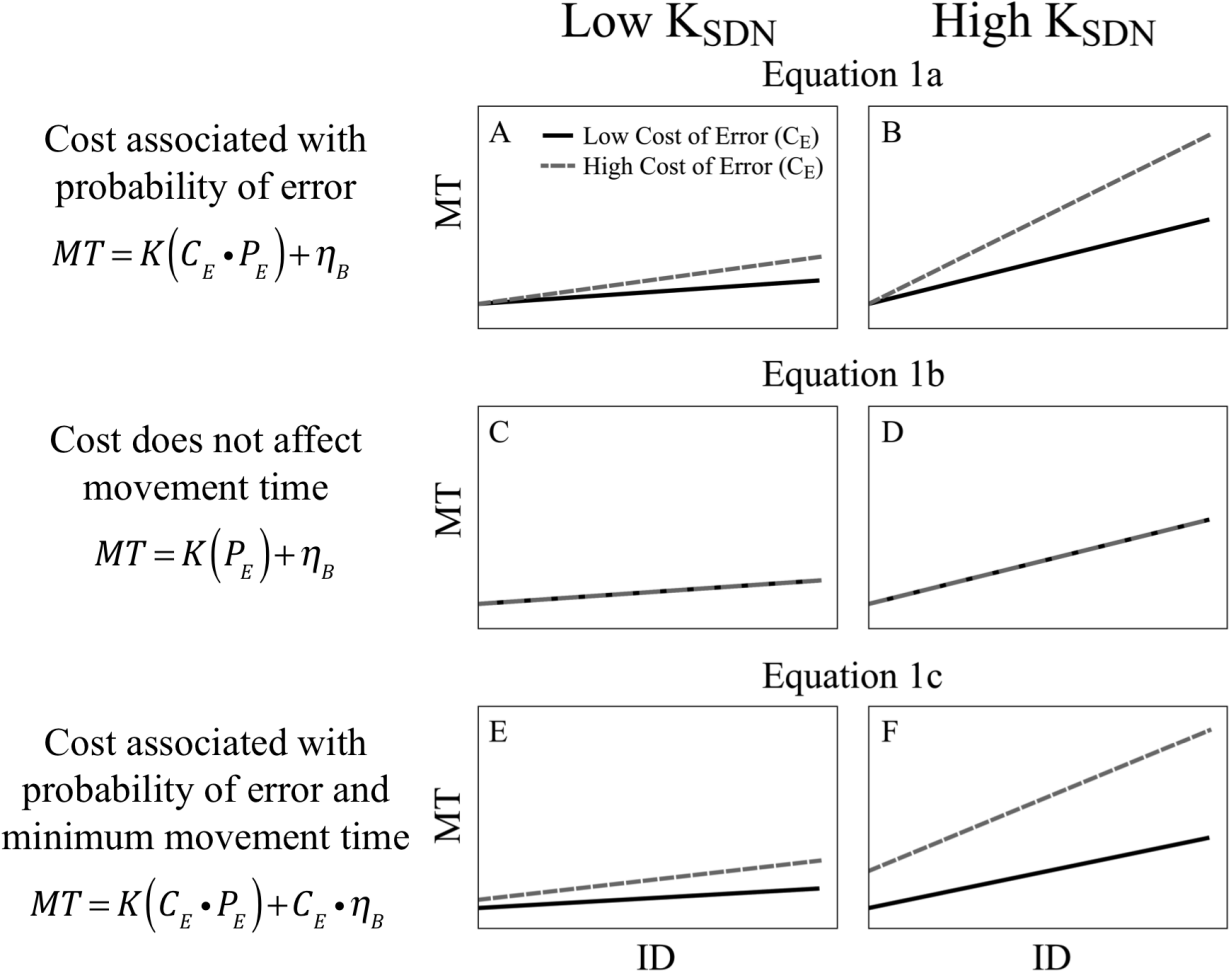
Model of cost and probability of error in the speed-accuracy trade-off. Theoretical model of predicted movement time (MT) over Index of Difficulty (ID) by taking into account the effects of different magnitude of signal-dependent noise (K_SDN_, left vs right panels) and cost of error at the target (C_E_, black solid vs grey dashed lines). Panels A and B predict that C_E_ would not affect the offset of Fitts’ linear relationship (constant η_B_), Eq 1; panels C and D predict that C_E_ would not have effects on MT, Eq 2; and in panels E and F, C_E_ would have effects on both the slope of the relationship between MT and ID and the offset (C_E_ x η_B_), Eq 3.

A risk-aware control framework has been recently proposed as a theory of motor control, which links ideas in optimal control with existing literature on risk behavior in humans [1]. Since the probability and cost of failure may vary throughout the workspace, the theory suggests that the nervous system estimates these values and plans appropriately. A recent study has shown that during a driving simulation game, humans modulate behavior based on both the cost and the probabilities of possible outcomes in response to environmental uncertainty [2]. Thus we predict that the movement time required to hit the target (MT) is a function of the cost and likelihood to miss the target:

$$MT=K\left(C_{E}\;↺P_{E}\right)+\eta_{B}$$ (1)

where K is a constant that translates the expected cost of error to the MT, C_E_ is the cost of error, and P_E_ the probability of error (missing the target). The constant η_B_ represents the minimum movement time to reach the target when P_E_ tends to zero, such as with large targets or small movement amplitude. This value is thought to be dependent on subject-specific properties of the sensorimotor system that are unrelated to noise, such as baseline levels of tone, co-contraction, overflow, and mechanical impedance (Fig 1A and 1B). Alternatively, it is possible that cost could have no effect (Fig 1C and 1D):

$$MT=K\left(P_{E}\right)+\eta_{B}$$ (2)

or that cost could be associated with both probability of error and the minimum movement time (Fig 1E and 1F):

$$MT=K\left(C_{E}\;↺P_{E}\right)+C_{E}\;↺\eta_{B}$$ (3)

According to the signal-dependent noise theory [4, 6, 19], P_E_ increases with either an increase in movement amplitude or a decrease in target size. Under this hypothesis, the scaling of MT with ID in Fitts’ Law is a strategy employed by the motor system to compensate for signal-dependent noise and minimize the probability of missing a target [4,7,20,21]. Furthermore, it has been shown that the magnitude of signal-dependent noise is also dependent on the state of the sensorimotor system; for example it may increase under muscular fatigue [22] or with pathological conditions of the central nervous system [17, 18]. We described P_E_ in the speed-accuracy trade-off framework as follows:

$$P_{E}=K_{SDN}\left(ID\right)$$ (4)

Where K_SDN_ represents a constant related to inherent signal-dependent noise and ID is the Index of Difficulty according to the Fitts’ Law formulation: log_2_ (2D/W). Thus, Eq 1 can be expressed as:

$$MT=K\left(C_{E}\;↺K_{SDN}\left(ID\right)\right)+\eta_{B}$$ (5)

In Fig 1, the panels on the left and right sides represent the prediction of MT as a function of ID with low and high signal-dependent noise (K_SDN_) respectively, predicted with Eq 1 (Fig 1A and 1B), 2 (Fig 1C and 1D) and 3 (Fig 1E and 1F).

In goal-oriented pointing movements, failing to hit a target results in a cost, i.e. negative value. The cost of missing the target can be represented in different ways, for example, the experimental design may include penalties (e.g. negative score), or repeating trials, which requires more effort. In addition to losing value due to missing the target, the performer squanders the benefit that he/she would gain from successfully hitting the target. Thus, the cost of error can be considered as C_E_ = C_M_ + C_P_, where C_M_ is the cost of the missed opportunity of success, and C_P_ is the penalty due to missing the target. We can rewrite Eq 5 as follows:

$$MT=K\left(C_{M}+C_{P}\right)\;↺K_{SDN}\left(ID\right)+\eta_{B}$$ (6)

The model predicts that higher signal-dependent noise (K_SDN_) increases the effect of cost of error (C_E_) on movement time. Thus, we hypothesize that children with dystonia (higher K_SDN_) will respond with a greater increment of movement time in a Fitts-like task (ID) under different cost of error (C_E_), due to the increase in risk. We also predict that the minimum movement time (η_B_) will not respond to changes in C_E_, because we believe η_B_ is based on fundamental properties of the sensorimotor system that are independent of cost. Thus, we anticipate that the intercept in the Fitts’ Law relationship will likely differ between groups (dystonic and control) but remain the same with changes in cost of error for each child.

## Materials and Methods

### Subjects

Sixteen children with a clinical diagnosis of either primary or secondary dystonia affecting one or both hands (13.7 years old ± 4.2 SD) and 15 healthy children (9.7 years old ± 2.5 SD) participated in the current study. The children with dystonia were recruited from the movement disorders clinic at Children’s Hospital of Los Angeles (CHLA). Their characteristics are outlined in Table 1.

**Table 1:** Characteristics of children with dystonia. BAD, Barry-Albright Dystonia Scale.

Participant	Age	Gender	Diagnosis	Left arm BAD score	Right arm BAD score	Preferred arm	Arm used for the task
P1	14	F	Primary dystonia; DYT1-	2	3	Left	Left
P2	18	M	Secondary generalized dystonia; vitamin E deficiency	1	1	Right	Right
P3	18	M	Secondary generalized dystonia; cerebral palsy	3	3	Left	Left
P4	7	M	Secondary generalized dystonia; cerebral palsy	1	1	Left	Left
P5	16	F	Secondary generalized dystonia; Glutaric acid urea type 1	3	3	Left	Left
P6	18	M	Primary dystonia; DYT1+	1	1	Left	Left
P7	11	M	Primary dystonia; DYT1+	0	1	Left	Right
P8	11	M	Secondary generalized dystonia; cerebral palsy	1	1	Left	Left
P9	16	F	Secondary generalized dystonia; right hemiplegia	0	3	Left	Right
P10	9	M	Primary dystonia; DYT1+	2	2	Left	Left
P11	11	M	Primary dystonia; DYT1+	3	2	Right	Right
P12	19	F	Secondary generalized dystonia; cerebral palsy	2	2	Right	Right
P13	9	M	Secondary generalized dystonia; cerebral palsy	2	2	Right	Right
P14	18	F	Secondary generalized dystonia; cerebral palsy	3	1	Right	Right
P15	16	M	Dystonic tremor	2	1	Right	Right
P16	8	M	Secondary generalized dystonia; cerebral palsy	2	3	Left	Left

Participants were excluded if there was clinical evidence of spasticity or corticospinal injury in the upper extremities, including hyper-reflexia, a spastic catch, or weakness. The University of Southern California Institutional Review Board approved the study protocol (IRB# UP-09-00263). Children’s parents gave informed written consent for participation, and all children gave written assent. Authorization for analysis, storage, and publication of protected health information was obtained from parents according to the Health Information Portability and Accountability Act (HIPAA). The study protocol was performed in accordance with the Declaration of Helsinki.

### Procedure

The experimental setup was the same as used previously [18]. Each participant attended a single experimental session of approximately one hour. All dystonic participants were previously rated on the Barry-Albright Dystonia scale (BAD) [23].

Participants sat in a chair or their own wheelchair in front of a table whose surface height was adjusted at the midpoint between the hip and the xiphoid process. They placed the hand that was not used for the task on their lap.

An iPad^®^ (Apple Inc, Cupertino, CA, USA) was located on the table in portrait mode in front of the participants at a distance that ranged between 40 to 55 cm. An adjustable metal bookstand supported the iPad^®^ to allow the participants a comfortable screen view. The size of the screen was 19.5 x 14.6 cm. Custom software was developed for the experimental task and distributed to the testing device via the Apple Inc. app store ("Bubbles-Burst" app, developed in the XCode 3.2 development environment, iOS 4.2 operating system; Apple Inc., Cupertino, CA, USA).

**Fig 2:**
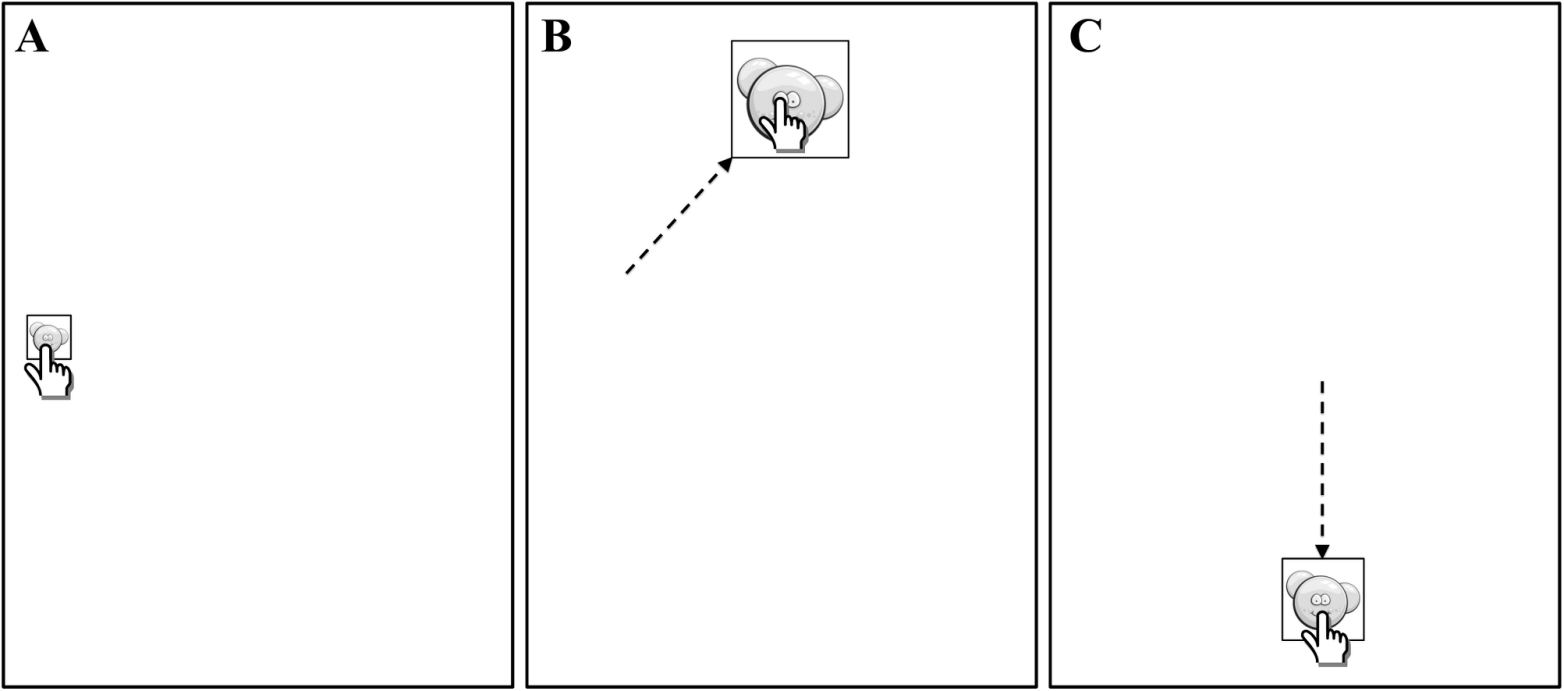
Task layout. A-B-C) A representative sequence of two consecutive trials with different target distance and target width with unspecified location of the subsequent target.

The subjects were required to touch targets on the iPad^®^ screen with the index finger of their preferred (less-affected) arm. Two children with dystonia (P7 and P9) were asked to perform the task with their non-preferred arm since the preferred was not affected by dystonia (BAD score equal to 0, see Table 1). Subjects were asked to maintain their trunk posture upright without touching the table or the bookstand with their pointing arm. Each trial was initiated by touching a 4 x 4 cm “start” button centered on the screen. Targets appeared at one of nine different locations on the screen, and subjects moved their finger sequentially from one target to the next. The targets were square with an image of three blue bubbles (Fig 2). Subjects were asked to touch and “burst the bubbles”. Subsequent targets appeared in a sequential manner on the screen at a random interval between 0.7 and 1.2 s and disappeared at the instant when the next one appeared. The subject was required to maintain contact with the last target until the next target appeared, without returning to the start button position. The location of the subsequent target was chosen pseudo-randomly. The study design consisted of 2 different experimental conditions: 1) a score penalty (Yes Penalty, YP) for missed targets and 2) no penalty (No Penalty, NP) for missed targets (see later in the methods for details). Note, YP and NP correspond to C_P_ > 0 and C_P_ = 0 in the theoretical model respectively, and C_M_ > 0 for both models.

**Fig 3:**
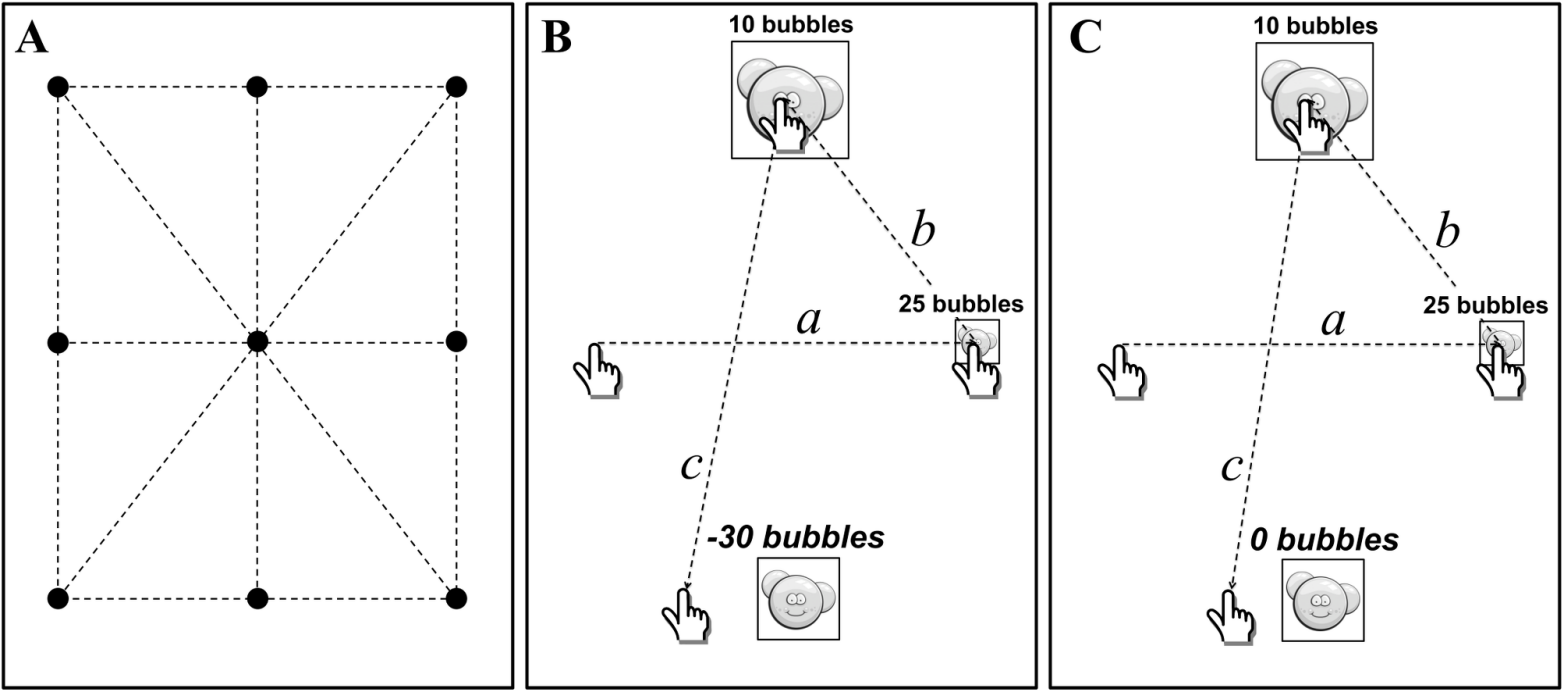
Target locations and penalty conditions. A) The nine locations of the targets on the screen (black circles) are shown; the dashed lines indicate the movement distances between targets. B) A representative sequence of three consecutive trials with different target distance and target width with score penalty for missed targets (Yes Penalty condition, YP). The score at each trial was based on the speed of the movement and the actual ID; note the values displayed in the figure were chosen only for demonstrative purpose. In case a target was missed, as the last move (c) shows, a 30 bubbles penalty was subtracted from the Total Score (TS, see methods) at the end of a block of 45 sequential targets. C) A similar representative sequence of three consecutive trials with different target distance and target width with none penalty for missed targets (No Penalty condition, NP). In this case, if a target was missed then no penalty was subtracted from the TS.

Each experimental condition (YP or NP) consisted of 180 targets divided into 4 blocks: 45 targets in each block with a one-minute interval following each set of 45 targets to avoid fatigue. In total, the participants performed 360 trials (180 x 2) divided into 8 blocks. The order of the two penalty conditions (YP or NP) was chosen randomly for each participant. For each penalty condition, six target distances (D = 4.5, 6.6, 8.0, 9.1, 13.2, and 16.0 cm) and three target widths (W = 1, 2, 3 cm) were used, yielding 18 different target conditions with different Indices of Difficulty, ID = log_2_ (2D/W) [5], varying from 1.60 to 4.99 bits. This resulted in 10 trials for each ID for each penalty condition. The locations of the targets were chosen to match the six experimental distances and the 18 target conditions (ID) were shown in a pseudo-random order throughout the 180 trials, which resulted in nine Cartesian coordinates displayed in a rectangular grid (Fig 3A). The software was programmed to avoid target locations that would be hidden by the hand or forearm at the start of the movement. It is worthwhile to notice that at the end of the four blocks within each penalty condition the subject had performed exactly the same target distances, widths and Indices of Difficulty.

A score, rewarded in number of bubbles, was visually provided at each touch, just above of the target, based on the movement time and actual ID. The subjects were told that the score was computed as follows: Score (S) = (2.5 x ID)/MT, where MT is the Movement Time, ID is the Index of Difficulty and 2.5 a constant term in order to return a tangible and reasonable score of performance.

At the end of each block of 45 sequential targets, the iPad^®^ displayed the Total Score (TS, [45 x S]) showing the “total number of bubbles” achieved. In the Yes Penalty condition (YP), a penalty of thirty bubbles points [e.g. “-30 bubbles”] was given for each missed target (Fig 3B). The penalties were, in that case, subtracted from the TS at the end of each block. In the No Penalty (NP) condition no bubbles [e.g. “0 bubbles”] were subtracted from TS in case of missed target (Fig 3C). Negative total scores in YP condition were possible at the end of each block. Before the test a practice block was performed consisting of two trials for each ID for both NP and YP conditions.

The actual instructions to the subjects were: “Touch and burst the bubbles as fast as you can to get the highest score, if you miss the target you will not lose bubbles”, or “Touch and burst the bubbles as fast as you can to get the highest score, if you miss the target you will lose thirty bubbles each time you miss” for NP and YP conditions respectively. Different auditory feedback was played for a hit or a missed target. Subjects were encouraged to maintain motion close to the plane of the iPad^®^ without an excessive displacement in the perpendicular direction. However, sliding the finger along the iPad^®^ to the next target was not permitted, and success required that the first contact with the finger on the screen be within the desired target. The entire experimental test required less than an hour for each subject.

The raw data collected from subjects has been made available as (S1 Dataset).

### Data Analysis

Custom software computed the Movement Time (MT) and the endpoint at the target for each movement. Later, the data were transferred to a laptop computer (MacBook Air, OS X, 10.8.4, Apple Inc., Cupertino, CA, USA) and analyzed using Matlab 7.10 software (Mathworks Inc., Natick, MA, USA). The MT was determined as the time interval between release of the index finger from the screen and the next contact with the screen. The location of the touch (and whether or not it fell within the target) was determined from the operating system pointer location.

The endpoint variability at the target (Var) was computed as the area of the ellipse containing the touch locations on the screen of each target width for each experimental condition with 95% confidence. This ellipse was obtained by applying Principal Component Analysis (PCA) to determine the direction of maximum and minimum dispersion of the distribution in the x-y plane. The eigenvectors of the covariance matrix were taken as the axes of the ellipse, while the lengths of the axes were determined by the corresponding eigenvalues [24, 25].

In order to test Fitts' Law, linear regressions were performed by the method of least squares for the averaged MT values across subjects within each group. The correlation coefficient was used to indicate the goodness of fit of MT (only successful trials) as a function of ID [26]. The intercept a of the linear regression equations and the fraction of successful touches (S = successful touches / 180) were calculated. We also computed the Index of Performance as the inverse of the slope b of the linear regression equations (IP = 1/b, [20]). The linear regression was also used to test the scaling effects of ID over (ΔMT).

We used a linear mixed-effects model to test the effects of group (Group factor: healthy children vs children with dystonia, random effect) and penalty condition for missed targets (Penalty factor; YP vs NP), and interaction effects for the variables S, intercept a and IP.

In addition to the Group and Penalty factors, we also considered two ranges of Index of Difficulty (ID factor; Easy: 1.59–3.17, mean = 2.55 and Hard: 3.19–4.99, mean = 3.99 bits/s) and target width (Target factor: small 1cm, medium 2 cm and large 3 cm;). We ran an independent sample t-test to test the difference between groups for the average change in movement time between penalty conditions (ΔMT) within each subject. Means (M) and standard deviations (SD) were computed for outcome variables. We used a criterion of P < 0.05 to signify a significant difference. The statistical analysis was performed using SPSS 16.0 (SPSS Inc, Chicago, IL, USA).

## Results

No subject withdrew from the study and there were no complaints of fatigue. No negative total scores were experienced in the YP condition in either group. We report statistical results for four independent measures: success rate (S), movement time (MT), index of performance (IP), and intercept (a). The linear mixed-effects model showed significant effects for Group and Penalty on success rate S [F(1,29) = 6.121, p < 0.05 and F(1,35) = 22.659, p < 0.001 respectively]. Children with dystonia had on average lower S (M = 0.85, SD = 0.13, 95% CI [0.81, 0.90]) than healthy children (M = 0.94, SD = 0.05, 95% CI [0.89, 0.98]). On average S with YP condition was greater (M = 0.92, SD = 0.09, 95% CI [0.89, 0.95]) than the NP condition (M = 0.87, SD = 0.12, 95% CI [0.83, 0.90]) considering both groups together. No significant interaction was observed between Group and Penalty.

**Fig 4:**
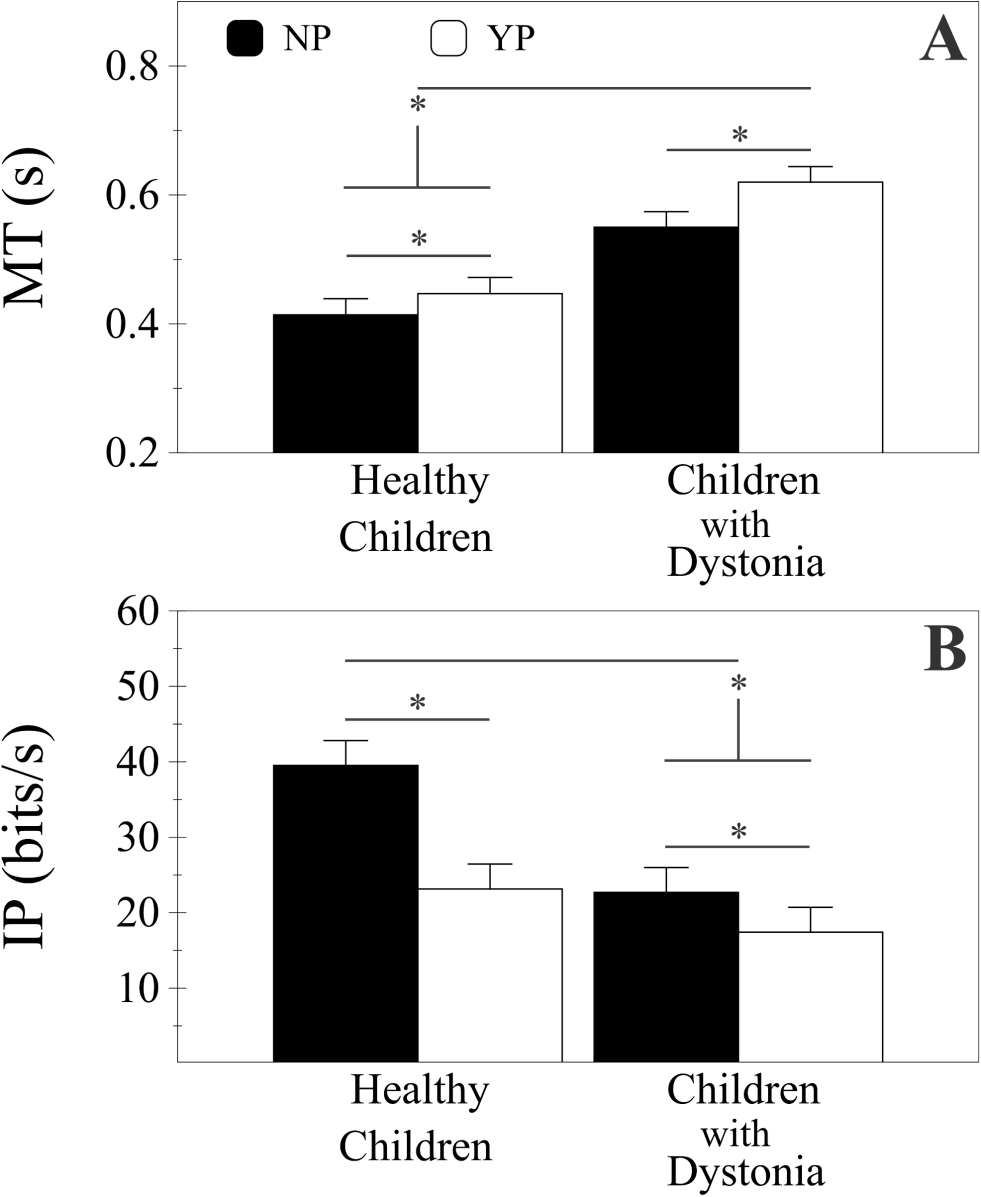
Movement time and Index of Performance. Plots showing the means and standard errors of the Movement Time (MT) (Panel A) and Index of Performance (IP) (Panel B) for both groups (healthy children and children with dystonia). Black bars: No Penalty condition, NP; white bars: Yes Penalty condition, YP. Asterisk mark (*) indicates a statistical difference p < .05.

There was a significant effect on MT for Group [F(1,29) = 20.728, p < 0.001], Penalty [F(1,1079) = 117.125, p < 0.001] and ID [F(1,1079) = 189.063, p < 0.001], with only significant interactions for Penalty and ID [F(1,1079) = 8.344, p < 0.01]. On average children with dystonia were significantly slower (M = 0.585 s, SD = 0.108, 95% CI [0.536, 0.633]) than healthy children (M = 0.430 s, SD = 0.094, 95% CI [0.380, 0.480]). On average MT was significantly less in the NP condition compared to YP (M = 0.482 s, SD = 0.111, 95% CI [0.447, 0.517] and M = 0.533 s, SD = 0.138, 95% CI [0.498, 0.568] respectively) and with Easy respect to Hard IDs (M = 0.475 s, SD = 0.125, 95% CI [0.440, 0.509] and M = 0.543 s, SD = 0.133, 95% CI [0.505, 0.576] respectively) considering both groups together (Fig 4A).

**Fig 5:**
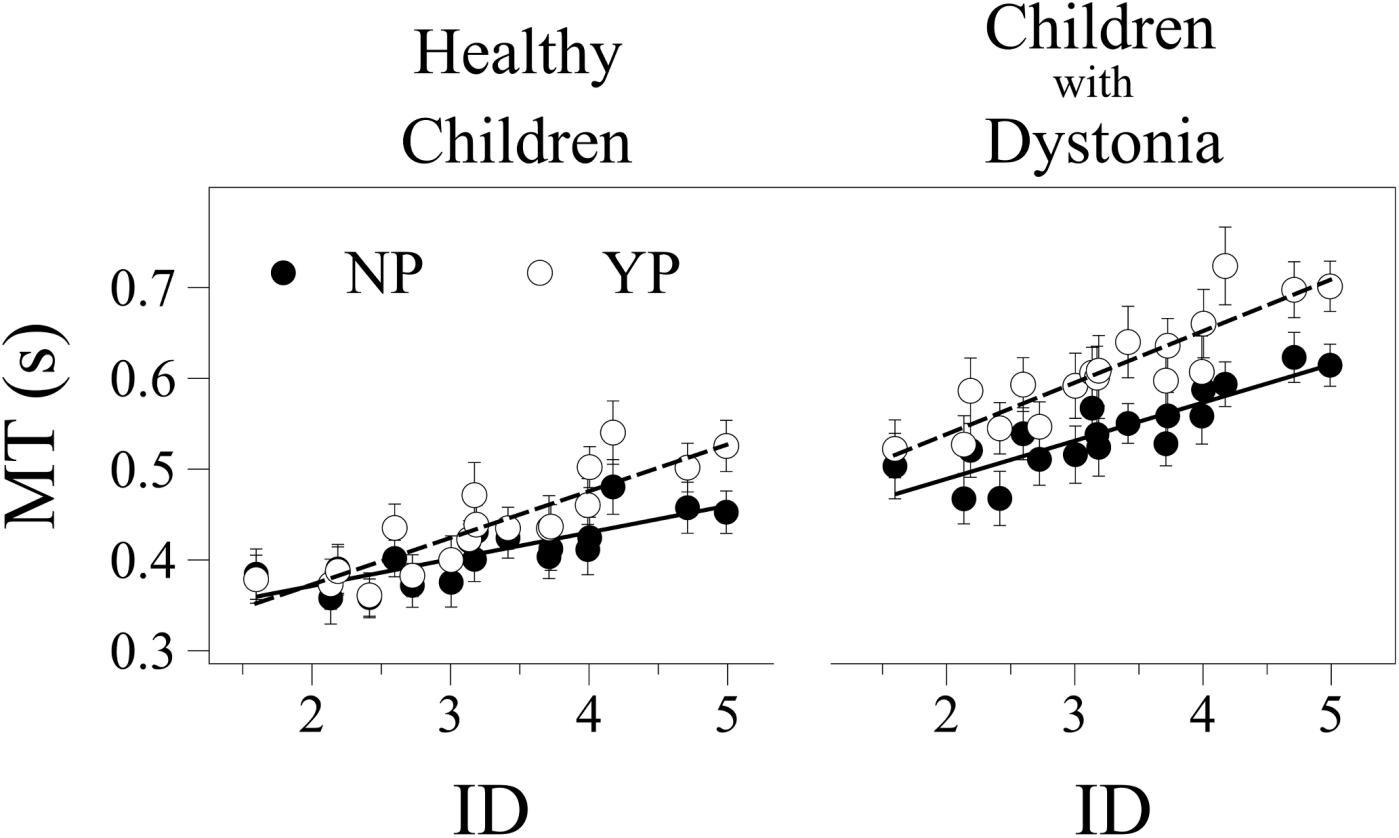
Fitts’ Law relationship. Mean movement time (MT) for both groups separated by penalty conditions (filled circles—No Penalty, NP; unfilled circles—Yes Penalty, YP) as a function of the Index of Difficulty (ID). The straight lines show the best fits by the least squares method.

MT showed a significant linear regression on ID for both groups on the two penalty conditions. In children with dystonia the correlation coefficients were 0.871 [F(1,17) = 50.487, p < 0.001] and 0.894 [F(1,17) = 63.694, p < 0.001] for NP and YP respectively. In healthy children the correlation coefficients were 0.800 [F(1,17) = 28.461, p < 0.001] and 0.876 [F(1,17) = 52.971, p < 0.001] for NP and YP respectively (see Fig 5).

There was a significant difference of intercept a between groups [F(1,29) = 7.735, p < 0.01]. On average children with dystonia had higher intercept in all conditions (M = 0.415 s, SD = 0.150, 95% CI [0.352, 0.478]) than healthy children (M = 0.291 s, SD = 0.110, 95% CI [0.226, 0.357]. There was not a significant difference of intercept a between NP and YP. No significant interactions were found.

IP showed a significant effect of both Group [F(1,29) = 9.069, p < 0.01] and Penalty factors [F(1,29) = 16.379, p < 0.001] with no interactions between Group and Penalty. On average, control children had a greater IP (M = 31.32 bits/s, SD = 10.42, 95% CI [25.83, 36.83]) than children with dystonia (M = 20.05 bits/s, SD = 10.42, 95% CI [14.72, 25.38]). Moreover, IP was affected by the Penalty for missing a target such that YP resulted in less information transmitted (M = 20.27 bits/s, SD = 12.81, 95% CI [15.66, 24.90]) than NP (M = 31.10 bits/s, SD = 12.81, 95% CI [26.49, 35.72]), see Fig 4B. There was a significant difference between groups for ΔMT [t(29) = -2.557, p < 0.05], and on average children with dystonia had a larger increase of MT with YP (M = 0.082 s, SD = 0.060) than controls (M = 0.029 s, SD = 0.054).

The pattern of results shown in Fig 5 resembles Fig 1A and 1B where healthy children are similar to the Low K_SDN_ group and children with dystonia are similar to the High K_SDN_ group. In Fig 1, both groups are modeled to have the same minimum movement time (η_B_), while in Fig 5, the dystonia group has a large movement time offset. Thus, it appears that both K_SDN_ and η_B_ are larger for children with dystonia as compared to healthy children. The lack of significant effects on the intercept between the penalty conditions NP and YP suggests that the model in Fig 1E and 1F is not the best match for the results in Fig 5. While we cannot rule out the possibility that cost is associated with minimum movement time, the lack of a significant difference suggests that this contribution to the intercept is relatively small compared to the cost associated with probability of error.

**Fig 6:**
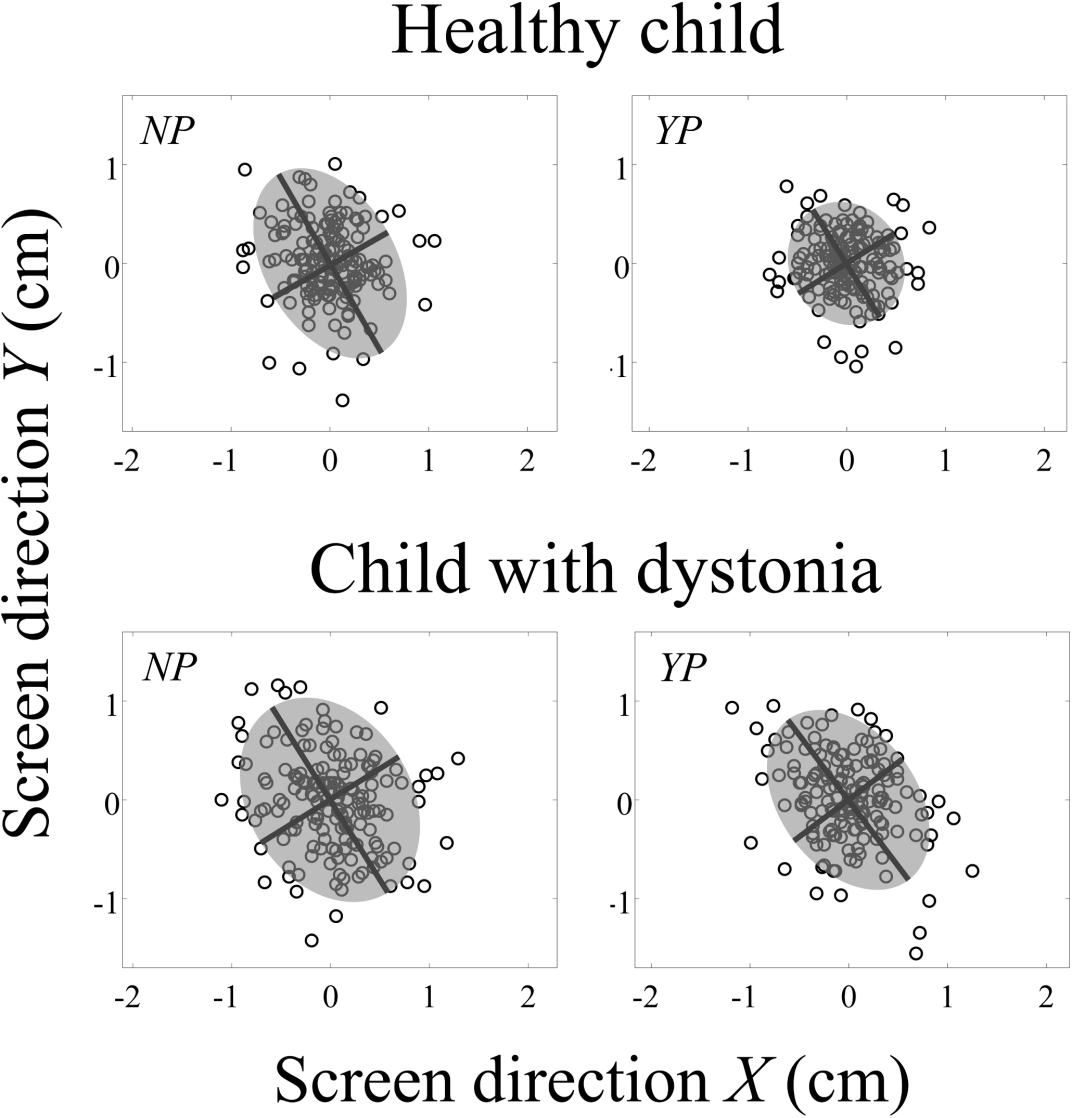
Representative endpoint distribution at the target. Endpoint distribution of the fingertip on the screen for both penalty conditions (No Penalty, NP; Yes Penalty, YP) in a typical healthy child (on the top panels) and patient P2 (at the bottom panels). The grey shaded areas circumscribe the ellipse defined by the two principal components of the dispersion of the distribution in the x-y plane on the iPad^®^’s screen. The black lines represents the eigenvectors of the principal components which were taken as the axes of the ellipse, while the length of the axes were determined by the corresponding eigenvalues.

Fig 6 shows the endpoint distribution of the fingertip on the screen for all four experimental conditions in a typical healthy child (right panels) and patient P2 (left panels). The grey shaded area circumscribes the ellipse defined by the two principal components of the dispersion of the distribution in the x-y plane on the iPad^®^’s screen. The black lines represent the eigenvectors of the principal components, which were taken as the axes of the ellipse, while the length of the axes were determined by the corresponding eigenvalues.

**Fig 7:**
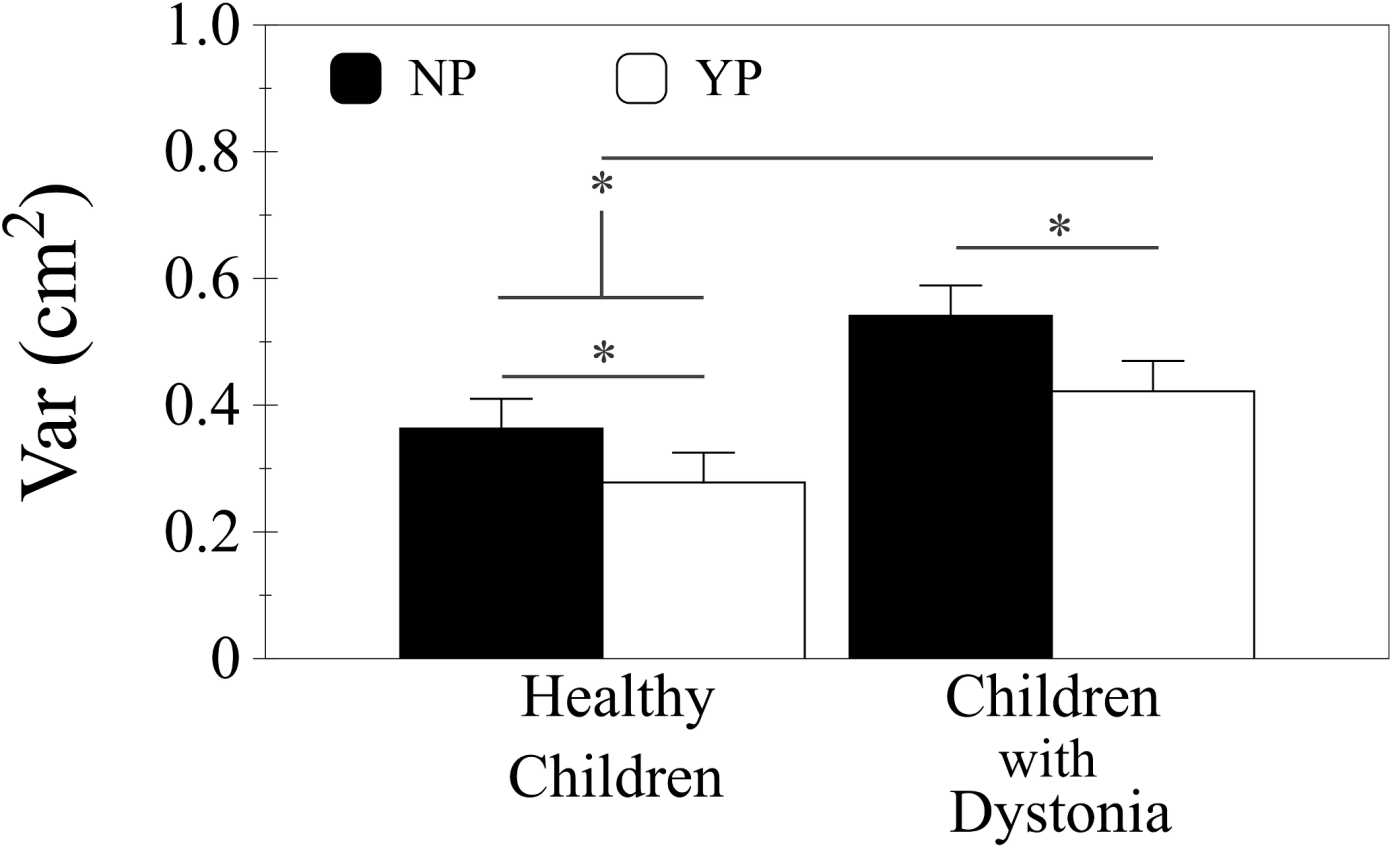
Results endpoint variability. Plot showing the means and standard errors of the endpoint variability at the target (Var) of penalty condition for both groups (healthy children and children with dystonia) for all the Indexes of difficulty (ID). Black bars: No Penalty condition, NP; white bars: Yes Penalty condition, YP. The asterisk mark (*) indicates a statistical difference p < .05.

There were significant effects for Group, Penalty and Target size on Var [F(1,27) = 5.269, p < 0.05, F(1,135) = 36.212, p < 0.001 and F(2,135) = 52.454, p < 0.001 respectively]; no significant interactions were observed between effects (Fig 7). On average Var was larger in children with dystonia (M = 0.481 cm^2^, SD = 0.301, 95% CI [0.378, 0.585]) than healthy children (M = 0.321 cm^2^, SD = 0.139, 95% CI [0.221, 0.421]). NP condition showed larger Var (M = 0.452 cm^2^, SD = 0.267, 95% CI [0.379, 0.526]) than YP (M = 0.350 cm^2^, SD = 0.211, 95% CI [0.277, 0.424]). The Pair-wise comparisons showed the largest Var for the big target (M = 0.511 cm^2^, SD = 0.246, 95% CI [0.435, 0.586]) than medium and small targets (p< 0.001, M = 0.394 cm^2^, SD = 0.242, 95% CI [0.319, 0.470] and p<0.001, M = 0.298 cm^2^, SD = 0.200, 95% CI [0.223, 0.374] respectively). Furthermore, the performances with the medium target resulted with larger Var than the small target (p < 0.001).

## Discussion

Risk is a ubiquitous feature of human movements, mostly due to unpredictable and unknown dynamics of the environment. We have defined risk as the Expected Cost, which is the product of the probability of error and the cost of error. The speed-accuracy trade-off is a fundamental principle of human motor control that may reduce the probability of error in attempting to hit a target [21]. The robustness of this principle (and its widely known mathematical formulation Fitts’ Law [7]) has been validated over the last 60 years across multiple motor behaviors and experimental conditions [21]. However, the recent consideration of motor planning and control of goal-oriented aiming movements within the framework of decision-making under risk suggests that the relationship between speed and accuracy may also be influenced when costs of erroneous outcomes vary [11–13]. We thus hypothesized that movement time in Fitts-like tasks emerges not only due the distance and size of the target (i.e. ID), but also due to the cost of errors. To test the hypothesis, subjects were asked to perform pointing movements with different costs of missing targets. We also manipulated the probability of error by comparing healthy children to children with dystonia, who are characterized by augmented signal-dependent noise. The findings of the study show a strong effect of cost of error on the Fitts’ law relationship with a proportional increase of the movement time with an increase of cost. Moreover, they show that increased inherent motor variability in children with dystonia is appropriately associated with greater sensitivity to the cost.

We have identified a close relationship between motor variability as measured by end-point variability (1.50 times higher for children with dystonia) and as measured by Index of Performance (1.56 times higher for children with dystonia). We believe that the same neurophysiological deficits underlie both manifestations of motor variability even though they are measured in different ways. Future studies on other populations with large movement variability (e.g. cerebellar ataxia [27]) will help determine if there is a direct relationship as we propose or if other factors are responsible.

The results are in line with the mathematical model proposed in which the movement time reflects the estimation of likelihood of error and cost of missing a target (Fig 1A and 1B).

While we have focused on explicit cost of error as the main cause underlying differences in movement time, it might also be due to greater implicit cost of error. Indeed the cost of missed opportunity in children with dystonia is smaller with respect to healthy children since, overall, they were slower and had lower scores, and so errors resulted in a lesser impact on their scores. There appeared to be a close connection between the relative motor variability of the groups and the relative index of performance (which is directly proportional to the explicit cost of error according to our model formulation). Thus, the parsimonious explanation is that variability is the main driver. However, we cannot rule out implicit cost, which we did not measure. Future studies aimed at distinguishing between explicit and implicit cost will help further understand differences between the populations.

### Fitts’ Law and probability of error

One theory of the origin of Fitts’ Law has been explained as a strategy to compensate for signal-dependent noise of the sensorimotor system [4]. Under this theory an increment of accuracy entails a decrement of speed in order to suppress the noise resulting from the motor command. Interestingly, the law can be seen within the framework of statistical decision theory [11, 13], such that a decrease of movement speed results in a greater likelihood to successfully hit the target (see also below). Indeed, it is worthwhile to note that in the original Fitts’ study [7] one of the essential experimental criteria was to control the error rate at the target (maintained below 5%). Only by maintaining a constant probability of error does Fitts’ Law emerge. Consequently, any increase of inherent motor variability could lead to changes in the Fitts’ relationship.

Recent work suggests that increased signal-dependent noise during motor execution describes the motor variability in childhood dystonia [17, 18]. The results of this study further corroborate the theory of augmented signal-dependent noise in dystonia. Children with dystonia had a wider endpoint distribution at the target than typically developing children in both penalty conditions, accompanied by a lower percentage of successful hits. Poorer speed-accuracy trade-off in Fitts-like tasks due to neurological disorders has been previously reported [28–30]. Thus, although the scaling between movement time and Index of Difficulty was preserved, children with dystonia showed an upward shifted effect on the Fitts’ linear relationship compared with typically developing children, with a lower Index of Performance. It is worthwhile to note that notwithstanding severe motor deficits, children with dystonia were able to perform the task with minimal errors at the targets (~15%). Therefore, to compensate for increased inherent motor variability, children with dystonia reduced movement speed appropriately in order to reduce the probability of missing the target. The Index of Performance describes a greater sensitivity to an increment in the Index of Difficulty that further explains their compensatory strategy as the probability of error increases.

We found a non-significant tendency for larger variance in the direction of the movement compared with the direction perpendicular to movement. Although the endpoint distribution, defined by PCA, was elongated in the direction of the movement, this tendency was not consistent across subjects, in particular for children with dystonia. Future work would be needed to investigate the effects of risky environments on spatial features of movement variability in speed-accuracy trade-off tasks.

To summarize, where the cost of error is kept constant, our findings show that Fitts’ Law emerges as expected, with an estimated interaction of task difficulty (i.e. ID) and inherent variability of the motor system in order to minimize the probability of error at the target.

### Movement time in Fitts-like tasks is related to risk

Recent work in motor control formulates movement planning in terms of statistical decision theory, essentially converting the problem of movement planning to decision-making under risk [10, 11]. In most cases, human subjects choose strategies that come close to minimizing the cost of behavior, for example maximizing expected gain [10] in motor tasks by combining noisy sensory input with prior information [31, 32], changing experimental stochastic variability [14, 33], and changing payoffs [13].

Recently the theory of “Risk-Aware Control” has proposed a new mathematical formulation in which movement planning and control are governed by estimates of risk based on uncertainty about the current state and the knowledge of cost of errors [1]. The theory allows for safe behavior in an unpredictable environment including in the presence of increased motor variability due to injured states of the CNS. An interesting prediction of the theory is that motor behavior will be modified by the perceived risk even if failure has not yet been experienced [1–2].

Our results are in accordance with previous studies that showed the ability of young adults to shift their mean points of contact with a computer screen in response to changes in penalty and location of penalty [13–15]. Recent results also showed that typically developing children and children with dystonia changed their movement strategies in response to changes in the level of perceived motor variability [33]. Our findings have shown that typically developing children and children with dystonia also demonstrate the ability to modulate their speed and movement variability in response to changes of cost of errors in the absence of any changes in the biomechanical and dynamical characteristics of the task.

Several interpretations have been offered for the intercept of the linear regression model in Fitts’ Law [34, 35]. Our findings suggest that intercept “a” may reflect components that are independent of distance and target size. Indeed, studies showed higher levels of muscle tone, co-contraction, and overflow as additional deficits of the motor system in childhood dystonia [36–38]. The independence of intercept “a” with the cost of error suggests an unrelated effect of motor planning with the offset of the Fitts’ model. In our model the intercept “a” is represented by the term η_B_.

The Index of Performance has been applied as a fundamental metric in quantifying information transmission in Human-Computer Interaction input devices [34, 39]. The impact that the perceived cost of missing targets has on the rate of information transmission while interacting with input devices may have widespread applications on designing touchscreen user interfaces for subjects who depend upon augmentative and assistive communication (AAC) technology.

Awareness of risk guides all of our actions, and this is essential for successful performance of motor actions in unpredictable environments. This study provides evidence that planning and control of Fitts-like tasks emerge from a weighted estimation of the cost of error and the likelihood of failure in order to minimize the risk of undesirable motor outcomes. Our results demonstrate that the cost of failure, together with signal dependent noise, affects the planning and control of movement in the speed-accuracy trade-off. The altered control plan could result in an overall reduction in muscular drive to reduce variability and/or alternative neuromuscular strategies may be used to respond to risky environments (e.g. muscle co-activation or higher co-contraction). To our knowledge, it is still unknown to what extent risk affects the choice of these neuromuscular strategies. Future studies that systematically measure changes in EMG as cost of error is manipulated will likely be able to distinguish between these possibilities. In particular, it will be interesting to determine how disease states impact the contribution of different strategies used by the central nervous system. For example, it is possible that children with dystonia tend to favor the cocontraction strategy while healthy children tend to reduce overall muscular drive.

The framework of decision-making under risk may provide new insights for understanding the underlying neurophysiological mechanisms of injured CNS states in which the control of movement or the perception of risk during motor planning are altered [16, 40–42].

## Supporting Information

S1 Dataset Raw data. Data is organized by penalty conditions (No Penalty and Yes Penalty) with subjects on the columns (1–15 Healthy Children and 16–31 Children with Dystonia) with the Indices of Difficulty and target size on the rows for Movement Time (MT) and Endpoint Variability (Var) respectively.
(TXT)
